# American Academy of Dental Science

**Published:** 1893-06

**Authors:** 

**Affiliations:** American Academy of Dental Science


### AMERICAN ACADEMY OF DENTAL SCIENCE.

THE regular monthly meeting of the American Academy of Dental Science was held in the Boston Medical Library Association rooms on January 4, 1893, at 7.30 P.M., President Brackett in the chair.

The paper of the evening was read by Dr. J. E. Waitt; subject, "Anæsthesia, and the Use of a New Ether-Inhaler."

(For Dr. Waitt's paper, see page 401.)

### DISCUSSION.

*President Brackett.*—With Dr. Waitt's co-operation, the subject is before you for discussion.

*Dr. Stevens.*—What do I understand becomes of the exhalations?

*Dr. Waitt.*—This valve being open, everything passes out here. In case it is desired to quickly force patients through the second stage of anæsthesia, it may be done by closing the valve and compressing the bulb rapidly. Later, the valve can be opened again and the process carried on more slowly.

*Dr. Eames.*—I would like to ask Dr. Waitt if, in the rapid induction of anæsthesia which may be brought about by this special apparatus, he has noticed a disturbance in the circulation, indicated by blueness of the lips and face.

*Dr. Waitt.*—I have not seen a single case of it with this apparatus. You can guard against it by closing the valve and forcing a larger amount of ether vapor into the inhaler.

*Dr. Fillebrown.*—Do you mean that you can overcome cyanosis by giving more of that which produces it?

*Dr. Waitt.*—No. I take it that cyanosis is produced by the patient not getting the requisite amount of air with the ether. In this apparatus the amount of air can be increased without removing the inhaler or stopping the application of ether.

*Dr. Fillebrown.*—Then, in compressing the bulb slowly you have a larger percentage of ether vapor than you do when pumping fast?

*Dr. Waitt.*—Yes. There isn't time for the air to take up so much ether when it is being forced rapidly.

*Dr. Fillebrown.*—Do you know what percentage of ether the air takes up at the rate at which the bulb is ordinarily compressed?

*Dr. Waitt.*—I do not. Three cubic inches of air are forced through the ether with each compression of the bulb, but I could not say what percentage of ether is taken up, either at the regular or at the rapid rate of compression.

This rubber tube can be made long and the bulb placed under your foot, so that your hands will be free. This little hook is convenient for handling the bottle in hospital practice. The apparatus can also be fastened to the side of your chair.

The inhaler is the invention of Dr. Horace Packard, of this city, and can be obtained at Codman & Shurtleff's or at the Boston Dental Manufacturing Company. The present price is fifteen dollars, but they hope by getting out a greater number to make it less expensive.

*Dr. Fillebrown.*—I have had experience with this inhaler in one case, and I am very much pleased with its action, especially because it avoids the cyanosis which so often follows the ordinary method of giving ether. The patient with whom I tried this method I had previously anæsthetized in the common way, and cyanosis was very noticeable, so much so that we had to suspend the ether and resort to artificial respiration.

This inhaler has been in use for some time by the inventor, Dr. Horace Packard, but it has never been in the market until within two weeks.

In the operation referred to above, Dr. Batcheldor, who was accustomed to the use of the inhaler, kindly administered the anæsthetic. The operation was staphylorrhaphy, and the patient was under the influence of ether for nearly an hour and a half. I had to stop frequently to have her reanæsthetized, but there was no cyanosis, and she recovered quickly from the anæsthetic and without nausea.

I feel very favorably towards the apparatus, and think it worthy the attention of those who wish to give ether or chloroform.

*Dr. Williams.*—Speaking of cyanosis reminds me of a little story regarding an apparatus that was presented some years ago to give a supply of atmospheric air. At that time the matter of anæsthesia was not fully understood, and in taking gas the patient would breathe into a bag and inhale from the same over and over again. An apparatus was devised which had an inspiratory and an expiratory valve, of which a descriptive circular came to me. I called on a person named in the circular, and asked him what his experience was with the apparatus. He said, "he had tried it a week or so and it had not worked very well." I then asked him, "Do you notice in using it that you have any blueness of the face or lips?" He said, "No; but he thought it was best to blue them a little; they were not so apt to feel it!"

*Dr. Banfield.*—I would like to ask Dr. Waitt if he uses gas in extracting.

*Dr. Waitt.*—I probably administer ether twenty times to gas once. I only administer gas for extracting one or two teeth, or in some operation not requiring much time. My preference is to use ether, and ordinarily, by carrying a person to the second stage of anæsthesia with this apparatus, you can extract the whole upper set of teeth without replacing the inhaler. Of course, if the teeth break easily and you have to dig for the roots, you could not finish the operation with one application of ether. But you have the advantage in using this apparatus that you are not wasting ether all the time, and it is not being distributed about your office, nor does the ether come in contact with the patient's face. In the average case of extracting, about half an hour would elapse from the time you commenced administering the ether until the patient was ready to leave the office. Out of one hundred tabulated cases where this apparatus was used, there were but two or three where any tendency to retching was observed, and those thus affected were etherized without paying attention to the time of eating.

*Dr. Briggs.*—I think this is a good thing. It secures a minimum amount of the drug and a proper admixture of air, and I don't think those two things have been so well combined in any apparatus before.

We have been accustomed to giving ether and chloroform in the most unscientific manner as compared with our care in the use of other drugs. We would not pour two or three ounces of laudanum into a man, and expect his system to absorb just enough of it and throw off the rest. That is practically what we have been doing in the old methods of giving ether, but here it is scientifically administered.

*Dr. Moffatt.*—I would like to ask Dr. Williams if he remembers the inhaler we used in Morton's time. I think Dr. Keep had one of them. It was rather bulky in construction, and the air entered only through the globe. Morton suggested that to force the anæsthesia you might plug up the orifice.

*Dr. Williams.*—He fixed it so you could "blue them a little." Yes, I remember it. He came near killing a classmate of mine with it; didn't give him air enough with the ether.

*Dr. Waitt.*—That was the original inhaler used by Morton.

*Dr. Briggs.*—I have been asked to supplement a paper on the use of cocaine in removing the pulp, which was read some time ago before the Academy. I will read you some of my cases treated last year.

The first case (January 2) happens to be the patient on whom I first tried the operation. Here was a tooth that had an exposed pulp which had been dressed with the desire to save it. The patient had neglected the tooth, and when she came to me again the pulp had become irritated, and I therefore injected cocaine, extracted the pulp, and filled the roots.

On January 14 I used this treatment on a sixth-year molar that I had been trying to save for six or seven years. The patient was a boy, and I had been waiting for the tooth to reach maturity so that I could put a cap on it. Having concluded that it had attained its full development, and that there was no need of longer keeping the pulp alive, I injected the cocaine, filled the roots, and prepared the tooth for a gold cap, which was put on a week later, and there has been no subsequent trouble.

The next case was a new patient, who came to me with an aching tooth from which she had been suffering for a week. I found an exposure, injected cocaine, extracted the pulp, and filled the roots at the same sitting. There was no trouble afterwards.

As I pointed out in the paper I read, you do not really kill the tooth, you simply remove the pulp.

The next case was also a new patient, who came in with the tooth-ache. When she left my office the roots of that tooth were filled. So it goes on. Fifty cases of removal of the pulp in a year perhaps sounds startling, but the majority were in new patients, who came with the toothache, and I have finished with the troublesome tooth then and there.

I will not take up more of your time, except to mention one case that was particularly successful. The patient came complaining of toothache, which was so intense that I could not touch the side of his face on account of the pain. With a good deal of trouble I made an examination, and found an exposure in the lower right third molar where the pulp had hypertrophied. I found this old syringe-point with a trumpet-shaped opening, which just capped over the little enlargement of the pulp. I injected the cocaine and took the pulp out, and then told the patient what I had done. I do not tell the patients what I am doing until I am all through, because it might make them nervous.

One day, while operating for a lady, I found that there was an exposed pulp, and knowing the tendency to neuralgia when there was any trouble with her teeth, I injected cocaine into the exposure, removed the pulp, and filled the roots, and then told her about it. She expressed great astonishment, and said that I had not hurt her at all.

The idea is to have the point of the syringe cover the opening into the pulp-chamber, so as not to press the syringe into the pulp.

The solution that I use is a twenty-per-cent. aqueous solution.

*Dr. Waitt.*—Can't you get the same result with a ten-per-cent. solution?

*Dr. Briggs.*—I cannot get it with anything less than a twenty-per-cent.; and another very important thing is that it must be fresh. The solution is good for nothing when it is at all old.

*Dr. Fillebrown.*—How long does the anæesthesia persist?

*Dr. Briggs.*—Not very long; you have to work quickly.

*Dr. Waitt.*—How long after using cocaine do you begin to work?

*Dr. Briggs.*—I don't wait at all. I take the engine and go right to work. Sometimes I make a second injection, so as to make sure that there will be no pain.

*Dr. Andrews.*—I would like to ask Dr. Briggs what broach he uses.

*Dr. Briggs.*—Donaldson's.

*Dr. H. F. Hamilton.*—After a careful, thorough, and conservative practice of the method of removing pulps proposed by Dr. Briggs, extending over two and one-half years, I regard it not only as a pleasure, but a duty to report that I consider it the greatest advance in dentistry that has been made in many years. It is distinctly new and novel in its operation, and leaves the tooth in a healthier condition than when arsenic has been used.

All who have tried this method will, I am sure, agree with me in this statement. I have used it lately only in favorable places and conditions, with the rubber dam, and under such favorable conditions I have had uniformly successful results.

There are three ways in which one can fail:

1. The difficulty of access to the cavity rendering it impossible to force the cocaine into the pulp.

2. The use of old cocaine.

3. The non-ability to expose the pulp and properly apply the cocaine. This may come from lack of expertness in the operator or from nervousness of the patient.

The remedies for all such failures are apparent, and I see no reason why the operation should not take rank as a sure and safe one. By this method one can easily remove the pulp and fill the canal at one operation, and its rapidity and simplicity offer a relief from the necessity of capping many exposed pulps. I have, after watching many cases of my own capping, come to the conclusion that in most cases of patients of mature years, and in the teeth back of the cuspids, it is better to kill the pulp and fill at once.

I consider that a half-worn cement or gutta-percha filling, letting food in against the gum and allowing the teeth to crowd together as they may, a very bad condition, and sure to bring extreme disasters in its train.

Again, I think that we have not as yet realized the trials to patient and operator of calcifying and calcified pulps. Dr. Cooke has ably shown us how common they are, and all of us have realized their obscure and annoying symptoms, and the extreme difficulty of diagnosis and treatment. From my experience and from conversation with others, I am led to call your attention and ask your observation in regard to the extent of calcification of pulps under capped exposures.

I consider that in the majority of cases the capping of pulps is merely inviting calcification, and where the conditions are favorable for injecting cocaine, I think it better practice to remove the pulp at once.

I wish to add a word of caution as to the use of twenty-per-cent. cocaine in the mouth. The rubber dam should always be applied, and if the tooth is decayed too far under the gum, a matrix should first be put on. In several cases where this could not be done I have sealed the cavity with soft oxyphosphate, applied the rubber dam, drilled into the pulp carefully on the buccal side near the gum, and injected the cocaine there. I then quickly removed the oxyphosphate and extracted the pulp through the cavity. A few breaths of ether will aid greatly in the drilling.

*Dr. Williams*—I came across an article in *Merck's Bulletin* for December, mentioning, as a new preparation, tropa-cocaine (or tropsin), which is an alkaloid recently isolated from a Java coca plant. Its advantages are:

1. It is less than half as toxic as cocaine.

2. Local anæsthesia, both of eye and skin, is much more quickly complete and is possibly of longer duration.

3. Its solutions are moderately antiseptic and retain their strength two or three months.

4. It anæsthetized in two minutes, and in one case in one-half a minute.

*Dr. Hamilton.*—I do not believe the members of the Academy realize the satisfaction to be obtained from this use of cocaine, and I advise you all to try it in the first favorable case.

*Dr. Fillebrown.*—Is there much hemorrhage?

*Dr. Hamilton.*—It is very slight; in some cases perhaps half a teaspoonful, and in other cases only a very slight quantity.

*Dr. Fillebrown.*—Not as much as when other agencies are used?

*Dr. Hamilton.*—Well, I don't know about that. I never tried any other agency for the extraction of a live pulp but once, and then I got only a piece of the pulp, but a great deal of experience.

*Dr. Andrews.*—I don't quite understand what is meant by the cocaine being forced in and around the pulp. Those of us who are familiar with the anatomical relation of the pulp to the dentine, as it appears under the microscope, know that it adheres to the walls of the tooth, and there is no chance for a fluid to get in between the two. I don't see in that case how you can "force it around the pulp."

*Dr. Briggs.*—I would not undertake to say that the cocaine is actually forced around the pulp, but that is the apparent effect. I mean to say that the best result is obtained by forcing it onto the pulp rather than into it. If your syringe-point happens to push into the pulp, you do not get the same result.

*Dr. Fillebrown.*—Is not the pulp compressible, Dr. Andrews?

*Dr. Andrews.*—To a certain extent; but if you cut a cross-section of a live tooth, you will see that the odontoblasts of the pulp are very closely connected with the tooth-walls.

*Dr. Fillebrown.*—Perhaps under the force used those little fibres are severed, and that may be a source of the anæsthesia.

*Dr. Grant.*—It seems to me that the actual force required to break down that connection would simply have the same effect as a steel broach.

*Dr. Fillebrown.*—The difference would be that the steel broach goes into the pulp, while the cocaine may remain on the outside and yet force its way between the pulp and the tooth-wall.

William H. Potter, D.M.D.,
*Editor American Academy of Dental Science.*

